# Disease Prevention versus Data Privacy: Using Landcover Maps to Inform Spatial Epidemic Models

**DOI:** 10.1371/journal.pcbi.1002723

**Published:** 2012-11-01

**Authors:** Michael J. Tildesley, Sadie J. Ryan

**Affiliations:** 1 Centre for Complexity Science, Zeeman Building, University of Warwick, Coventry, United Kingdom; 2 US National Institute of Health, Fogarty International Center, Bethesda, Maryland, United States of America; 3 Department of Environmental and Forest Biology, College of Environmental Science and Forestry, State University of New York (SUNY-ESF), Syracuse, New York, United States of America; Pennsylvania State University, United States of America

## Abstract

The availability of epidemiological data in the early stages of an outbreak of an infectious disease is vital for modelers to make accurate predictions regarding the likely spread of disease and preferred intervention strategies. However, in some countries, the necessary demographic data are only available at an aggregate scale. We investigated the ability of models of livestock infectious diseases to predict epidemic spread and obtain optimal control policies in the event of imperfect, aggregated data. Taking a geographic information approach, we used land cover data to predict UK farm locations and investigated the influence of using these synthetic location data sets upon epidemiological predictions in the event of an outbreak of foot-and-mouth disease. When broadly classified land cover data were used to create synthetic farm locations, model predictions deviated significantly from those simulated on true data. However, when more resolved subclass land use data were used, moderate to highly accurate predictions of epidemic size, duration and optimal vaccination and ring culling strategies were obtained. This suggests that a geographic information approach may be useful where individual farm-level data are not available, to allow predictive analyses to be carried out regarding the likely spread of disease. This method can also be used for contingency planning in collaboration with policy makers to determine preferred control strategies in the event of a future outbreak of infectious disease in livestock.

## Author Summary

Mathematical models of infectious diseases are increasingly used to inform policy decisions. The advantages of such models are that multiple control options can be rapidly tested and compared, without the risks and costs associated with field experiments. However, for such models to be practically useful tools detailed data (both in terms of populations and epidemiology) are required. In many countries, such as the USA, individual-level demographic information on livestock farms is generally lacking. However, remotely sensed information (such as satellite images and land-use maps) provides the potential to generate these data or produce surrogate populations. In this paper we use land cover data to predict farm locations in the UK and investigate the effect of a precise knowledge of farm locations upon epidemiological predictions in the event of a foot-and-mouth disease epidemic. Our results show that, when highly resolved land cover data are used to predict farm locations, accurate predictions of epidemic sizes, durations and preferred intervention strategies can be obtained. This suggests that land cover data may be used in countries where individual farm-level data are not available, to allow for analyses to be carried out regarding the likely spread of disease in future outbreaks.

## Introduction

Mathematical models of infectious diseases are increasingly used to inform policy decisions. The advantages of such models are that multiple control options can be rapidly tested and compared, without the risks and costs associated with field experiments. However, for such models to be practically useful tools detailed data (both in terms of populations and epidemiology) are required. In many countries around the world, detailed demographic data on livestock farms, wildlife populations or vector densities are generally lacking. However, remotely sensed information (such as satellite images and land-use maps) provides the potential to generate these data or produce surrogate populations.

In early 2001, an outbreak of foot-and-mouth disease (FMD) occurred in the United Kingdom for the first time in over thirty years [1]. A nationwide ban on animal movements was introduced for the duration of the epidemic to minimise spread of disease and mathematical modelers were consulted to provide policy advice to help control the epidemic [2]–[3]. The nationwide movement ban occurred in late February 2001 four days after the first case was reported. Whilst a movement ban reduces the risk of large scale disease spread, it does not prevent spread from occurring, as the virus can be spread between farms via contaminated machinery, between infected livestock over farm boundaries, contaminated milk tankers, on people's shoes etc. In 2001, nearly 2000 farms were infected after the movement ban was introduced and the epidemic lasted over 7 months.

The ability of mathematical models to make accurate predictions in 2001 was facilitated by the availability of highly resolved demographic and infection data. In the UK, an annual livestock census records the location and species composition of all livestock farms in the UK (though these livestock quantities are subject to some variations throughout the year). During 2001, the Disease Control System (DCS) database recorded the location, reporting and cull date of all infected farms, as well as detailed information regarding premises whose livestock were culled as part of the disease control effort [1], [4].

These detailed databases have allowed for retrospective statistical analyses of the 2001 epidemic [5]–[7] and modeling of preferred control policies both for the 2001 epidemic [4], [8]–[9] and future outbreaks of disease in the UK and elsewhere [10]. However, whilst data availability is good for the UK and other countries in Europe, the same is not true for many other countries around the world. In the USA, an agricultural census is carried out by the National Agricultural Statistics Service (NASS) every five years. Whilst this database is available in the public domain, there are strict laws in place regarding the types of data that the Federal government can collect and maintain. This includes any data regarding precise locations of livestock farms and therefore the NASS data are aggregated at the level of the county in order to assure anonymity for farmers. Large value is placed upon personal freedoms in the USA, including the right of the individual to privacy, and in the current climate, it seems unlikely that farm locations would ever be made available to disease modellers. Should there be a major disease outbreak, there may be pressure to release these data, but it is vital to have models in place to use at the onset of an epidemic – by the time that precise location data may be available, it could be too late for models to have any effect upon reducing disease spread and advising strategies for disease control.

In general, in the event of an outbreak of disease, the risk of infection of susceptible individuals decreases with distance from any source of infection, with spatial structure playing a key role in disease transmission for both humans [11] and animals [2]–[3], [12]–[13]. In the case of FMD, the main risk factor contributing to transmission of disease has been shown to be proximity to previously infected farms [14]–[16] and it therefore seems that a precise knowledge of farm locations may be vital to making epidemiological predictions.

Previous work for both the UK and US has investigated the ability of county-level models with random farm locations to predict the spatio-temporal dynamics of future epidemics [17]. The results show that, whilst random location models generally underestimate epidemic sizes, preferred control policies (specifically ring culling) are found to be not substantially different for both the random location and recorded farm location data models. However, there is an important caveat to this. In this analysis, the random location model provides robust policy advice only when parameterised to fit early epidemic data, indicating that random location models such as these could only be used once an epidemic is in progress. In addition, whilst a model which can accurately predict control strategies to minimise spread of disease has obvious merit, one which can also predict the likely size and duration of the epidemic would be highly beneficial for policy planning purposes.

This paper aims to build on the analysis of [17], by investigating the ability of land cover data to usefully predict farm locations. The spatio-temporal dynamics of a potential future outbreak of foot-and-mouth disease in the UK and the impact of intervention strategies as predicted by these synthetic databases are explored and compared with observed outbreaks on the recorded farm location data. Whilst previous work has investigated how landscape heterogeneity influences outbreaks of disease [18]–[19] and abundance of infected hosts [20]–[22] this is, as far as we are aware, the first investigation into the ability of land cover to make accurate disease control predictions in the absence of knowledge of precise locations of individuals (farms). The novel analysis presented in this paper will therefore provide useful insights into our ability to use land cover data to approximate farm locations in countries such as the USA where precise data are not available, and enable the development of models to be used in the event of future outbreaks of disease.

## Materials and Methods

### The model

The mathematical model used throughout this paper is an adapted version of the model first used by Keeling and coworkers [2] to predict spread and optimal control strategies during the course of the 2001 FMD outbreak, after the introduction of a nationwide movement ban. Farms are classified as susceptible, exposed, infectious, reported or culled. A susceptible farm can catch foot and mouth disease (FMD) from any infectious farm, after which it is classified as exposed (the animals are incubating the disease but are not yet infectious). The farm then transitions into the infectious class and disease can now spread to susceptible farms. In general, symptoms of the disease appear about 9 days after infection, after which the farm becomes reported and the farm is prioritised for culling. In line with previous estimates [4], [9], [10], [17], we assume an exposed period of 5 days, an infection-to-report period of 4 days and a report-to-culling delay of 1 day (see below).

The probability that a given susceptible farm *j* is infected by any infectious farm *i* on each day is given by:
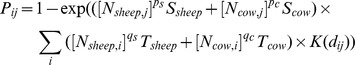
N_s,i_ is the number of livestock species *s* recorded as being on farm *i*, *S_s_* and *T_s_* measure the species-specific susceptibility and transmissibility, *d_ij_* is the distance between farms *i* and *j* and *K(d_ij_)* is the distance-dependent transmission kernel, estimated from contact tracing [2]. Finally, the parameters, *p_s_*, *p_c_*, *q_s_* and *q_c_*, are power law parameters accounting for a non-linear increase in susceptibility and transmissibility as animal numbers on a farm increase and provide a closer fit to the 2001 data than when these powers are set to unity [5], [7], [10]. The model is therefore a discrete time Markov process where the probability of a susceptible farm being infected (and farms being culled or vaccinated) on a given day is dependent upon the state of all farms in the neighbourhood of that farm on the previous day. Once the infection process has been carried out, farms are updated into their new classes and the process is repeated.

Pigs were the infected species on only 18 farms in the UK 2001 epidemic and therefore we have little information as to the susceptibility or transmissibility of pigs for this strain of FMD, and are therefore not considered in the analysis. However, the effect of a potential future outbreak of FMD in the UK pig population has been investigated in detail previously [10].

In addition to culling of infected premises (IPs), all dangerous contacts (DCs), defined as “premises where animals have been in direct contact with infected animals or have, in any way, become exposed to infection”, were pre-emptively culled in an effort to control disease. DCs in our model are determined based upon both prior infection by an IP and future risk of infection in the same way as in previous work [9]. In 2001 there was a target of culling all IPs within 24 hours of reporting infection and associated pre-emptive culling within 48 hours. Whilst this result was rarely achieved in practice during 2001, we assume a 24/48 hour policy here. Previous work has investigated the effectiveness of this strategy compared with one using the culling delays actually carried out during 2001 [4].

In the event of an outbreak of FMD in the USA, it is assumed that culling of IPs and DCs would be carried out automatically and other control policies, such as ring culling and vaccination would be considered as additional intervention strategies. In the UK, vaccination was considered in 2001 but dismissed for a number of reasons. At the time, any livestock that were vaccinated would have to have been subsequently culled in order for the UK to regain its international trading status of “free from foot-and-mouth disease- without vaccination” status, and therefore the cost of carrying out a nationwide vaccination campaign would have been prohibitive. Since 2001, the Department of the Environment, Food and Rural Affairs (DEFRA) has introduced a vaccinate-to-live policy as part of their contingency plan in the event of future FMD outbreaks. The OIE (Organisation International des Epizooties – the international animal health standard setting body) has defined rules for countries to recover their disease free status. In the event of vaccination-to-kill, disease free status can be regained three months after the slaughter of the last vaccinated animal, whilst in the event of a vaccinate-to-live policy, disease free status resumes six months after the last reported case or the last vaccination (whichever is latest), provided that serological surveillance demonstrates that the remaining vaccinated population is not infected. It is unlikely that preventative vaccination would be considered as any country introducing such a policy would lose their “disease free without vaccination” status and this would heavily impact their export market. Also, cattle only retain immunity from infection for a few months (up to around a year) so repeated preventative vaccination campaigns would have to be administered, at significant cost. Finally, there are multiple serotypes of FMD virus with little to no cross-protection from a vaccination for one serotype. All of these factors would make a preventative vaccination campaign unviable in countries that are currently disease free without vaccination.

In this work, we therefore investigate the effectiveness of introducing a policy of ring culling or reactive vaccination in addition to IP and DC culling. We assume that resources to carry out control are limited and that a maximum of 100 farms can be ring culled per day and 35,000 animals can be vaccinated per day in line with previous work [9]–[10]. Sensitivity to these limits on vaccination and ring culling have been investigated elsewhere [9]–[10]. When an IP is reported, all farms within a particular radius of that IP will be targeted for culling or vaccination. The radius of the ring is allowed to vary between simulations and we seek the radius which minimises the “Epidemic Impact”, defined as the total number of farms with livestock culled (either as IPs, DCs or ring culled farms). We assume a “vaccinate-to-live” policy, such that uninfected vaccinated animals do not contribute to the overall Epidemic Impact.

Vaccination is assumed to take place within a ring around each IP such that all farms within a given distance of every reported IP will be vaccinated. Farms are vaccinated in the order they are identified and vaccination around each farm is performed from the outside in. The limit on the number of animals that can be vaccinated per day means that some farms will be vaccinated several days after they are first prioritised for vaccination. In line with previous work, a vaccine efficacy of 90%, a five day delay from the first reported case to the introduction of a vaccination campaign and a four day delay from vaccination to immunity are assumed. The number of animals vaccinated per day, vaccine efficacy, the time delay to vaccine introduction and the time delay to immunity will all influence the effectiveness of any vaccination campaign. Whilst we do not test sensitivity to these assumptions in this paper, their effect has been investigated in detail in previous work [9]. Throughout this paper 5 farms in a 10 km cluster are seeded initially with infection in each county prior to the introduction of a ban on animal movements, to approximate a localised outbreak, and these are defined as the primary cases.

### Data

The model is seeded using data from the agricultural census carried out in June 2000. Whilst the main aim of this paper is to determine the effect of a precise knowledge of farm location upon epidemiological predictions, it is also important to investigate the effect of farm size and composition at the individual farm level, as this information is also not available for many countries (including the USA). It may be possible to obtain summaries of farm information at the county or municipal level in some countries, thus it is important to assess how transferrable this type of model is for epidemic management. We therefore simulate these types of data aggregation and averaging to compare to our known data.

We use data from the Agricultural Census for four counties in the UK: Cumbria, Devon, Clwyd and Aberdeenshire. These four counties are chosen owing to their differing demographies and locations, allowing for a representative analysis of this approach across the UK. According to the June 2000 Agricultural Census there were 8036 farms in Cumbria, 11177 farms in Devon, 3563 farms in Clwyd and 3086 farms in Aberdeenshire. In the data sets “Shuffle”, farm sizes and species compositions are shuffled between farms within each county whilst farm locations are preserved. This, in a sense, enables a preservation of farm anonymity, whilst retaining the size and composition heterogeneity across the data. In the data set denoted “All Equal”, we assume precise farm locations are known, but only the total number of cattle and sheep within each county are known – as is the case in the public realm in the USA. Each farm is therefore assumed to have the same numbers of livestock, given by the average farm size within each county. This data set allows for an investigation into the effect of farm size heterogeneity upon disease dynamics. The data sets denoted “Shuffle CSM” – where CSM is Cattle, Sheep, Mixed - assume that the total numbers of farms that are cattle only, sheep only or mixed farms are known, in addition to farm locations and the total number of cattle and sheep within each county. Farm locations are therefore shuffled whilst farm sizes in each category (cattle only, sheep only and mixed) are identical, and given by the average livestock numbers for that category. These data sets will preserve clustering between farms but not necessarily between farm sizes and types. Finally, the data sets “Random” randomly locate farms within each county, thus removing all elements of clustering, whilst maintaining individual farm sizes.

In order to carry out an analysis of the effect of imprecise knowledge of farm location upon epidemiological predictions, we use land cover data compiled in 2000 as supplied by Land Cover Map 2000 (LCM2000) to create surrogate farm databases for the four counties in the UK listed above. LCM2000 categorises land within the UK according to land cover classes at two scales: 10 aggregate classes (AC) and 26 subclasses (SC) (see table 1). These land cover types are based on classification of satellite imagery from Landsat TM (thematic mapper), ETM (enhanced thematic mapper) and the LISS (linear self-scanning sensor) imagery, calibrated with field surveys, to develop nested ‘broad habitat’ (roughly corresponding to AC) and ‘priority habitat’ (corresponding to SC) maps in support of the UK Biodiversity Action Plan, the UN Convention on Biological Diversity and to support conservation agencies in the UK (for details and methods, see [23]–[24]). In the event of an epidemic in livestock in a country such as the USA where detailed farm data are not widely available, land cover may be used to approximate locations. LCM2000 enables us to investigate the effect of using such geographic information for the UK livestock system, as a means of comparing with known locations.

**Table 1:** Table listing all subclasses (SC) and aggregate classes (AC) in the 1 km^2^ LCM 2000 database.

SC No.	AC No.	SC Type	AC Type	Land Cover data set
1	10	Sea/Estuary	Oceanic Waters	
2	8	Water (inland)	Standing open water	
3	9	Littoral rock	Coastal	
4	9	Littoral sediment	Coastal	
5	9	Saltmarsh	Coastal	
6	9	Supra-littoral rock	Coastal	
7	9	Supra-littoral sediment	Coastal	
**8**	**6**	**Bog (deep peat)**	**Mountain, heath, bog**	**LC2**
**9**	**6**	**Dense dwarf shrub heath**	**Mountain, heath, bog**	**LC2, LC4**
**10**	**6**	**Open dwarf shrub heath**	**Mountain, heath, bog**	**LC2, LC4**
**11**	**6**	**Montane habitats**	**Mountain, heath, bog**	**LC2, LC4**
12	1	Broad-leaved/mixed woodland	Broad-leaved/mixed woodland	
13	2	Coniferous woodland	Coniferous woodland	
**14**	**4**	**Improved grassland**	**Improved grassland**	**LC1, LC2, LC3, LC4**
**15**	**5**	**Neutral grass**	**Semi-natural grass**	**LC1, LC2, LC3, LC4**
**16**	**5**	**Setaside grass**	**Semi-natural grass**	**LC1, LC2**
**17**	**5**	**Bracken**	**Semi-natural grass**	**LC1, LC2**
**18**	**5**	**Calcareous grass**	**Semi-natural grass**	**LC1, LC2**
**19**	**5**	**Acid grassland**	**Semi-natural grass**	**LC1, LC2**
**20**	**5**	**Fen, marsh, swamp**	**Semi-natural grass**	**LC1, LC2**
21	3	Arable cereals	Arable and horticulture	
22	3	Arable horticulture	Arable and horticulture	
23	3	Arable non-rotational	Arable and horticulture	
24	7	Suburban/rural development	Built up areas/gardens	
25	7	Continuous urban	Built up areas/gardens	
26	6	Inland bare ground	Mountain, heath, bog	LC2

The subclass number, the corresponding aggregate class (AC) number and the land types are listed. The final column indicates which of the subclasses are included in the generation of the data sets LC1–LC4. All ACs and SCs that are considered to be correlated with livestock farm locations are highlighted in bold.

We approach our land cover class selection as policy makers might - using the best information available, in terms of land cover, but without the resources to do exhaustive ground truthing for farm locations. To explore how this best information approach is vulnerable to decision maker opinion, we looked at two sets of landcover class selections at each scale. In the UK, the majority of cattle and sheep are kept on grassland (or housed, in the case of cattle). We therefore deduce that farms would be located in AC categories 4 and 5: “Improved grassland” and “semi-natural grass” respectively (see table 1). However, some (often small) sheep farms are also kept at higher altitudes. Thus, for a second broad scale land cover selection, in addition to AC categories 4 and 5, we may conclude that AC category 6, or “Mountain, heath and bog” would be a viable region in which farms may be located (see table 1). We denote these first two selections Land Cover 1 (LC1) and Land Cover 2 (LC2), in which farms are located within AC categories 4 and 5, and 4, 5 and 6 respectively.

At the finer scale, using LCM2000 to define likely farm locations based upon the subclasses (SC) within aggregate classes, we created a second, nested set of land cover selections, using more specific data. Land cover class AC 4 is comprised of only one subclass category (14, or “Improved grassland”), whilst AC 5, “semi-natural grass”, comprises 5 subclasses. Of these 5 subclasses only “Neutral grass” (SC 15), sounds likely to be correlated to livestock farm locations, while others such as “Fen, marsh, swamp” (SC 20) or “Bracken” (SC 17), do not (table 1). Thus, data sets defined by LC3 (Land Cover 3) are more highly resolved versions of LC1, in which farms are located only within subclasses 14 and 15.

AC region 6 is made up of 4 subclasses, of which “Dense Dwarf Shrub Heath”, “Open Dwarf Shrub Heath” and “Montane habitats”, or SC classes 9, 10 and 11 are deemed more likely to be correlated to hill sheep farm locations than “Bog” (SC 8) and “Inland bare ground” (SC 26). LC4 is therefore a more resolved version of LC2, comprising subclasses 9, 10, 11, 14 and 15. All aggregate class and subclass land cover categories deemed to potentially correlate with livestock farm locations are listed in table 1.

Surrogate farm databases are now constructed using these land cover values. The first spatially randomized data set “Random” simply distributed points across the landscape of each county, regardless of land cover. We then added geographic information by creating spatially randomized data sets constrained by land cover class (LC) selection, according to LC 1–4 (R scripts are provided in Datasets S1 and S2). Data points were randomly located within each land cover spatial data set within each county, corresponding to the number of farms in each of the four counties. The individual attributes of farms such as number of sheep and cows, and the area of the farm were then reassigned to the points. One hundred unique data sets were generated for epidemic simulation for each data type, with the exception of the data set “All Equal” (where only 1 data set can be generated for simulation).

## Results

**Figure 1:**
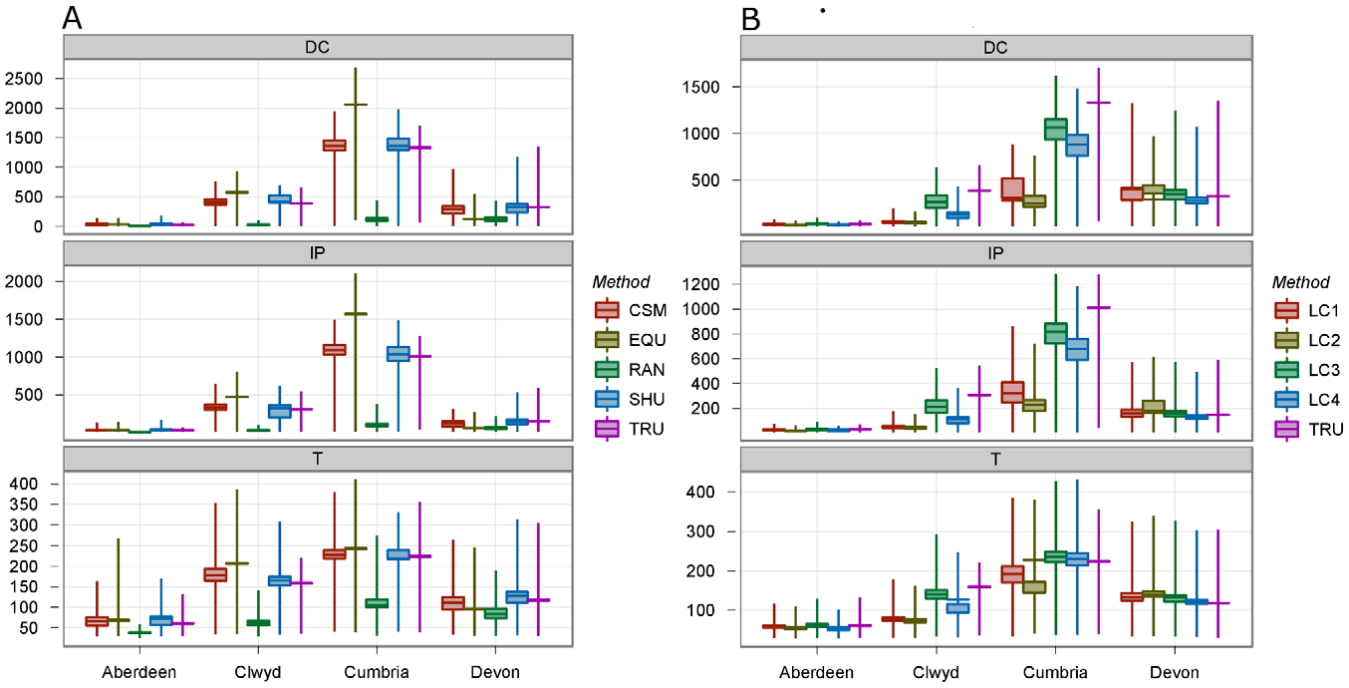
These plots show the number of dangerous contacts (DC, top panel), infected premises (IP, middle panel) and duration of outbreak (T, bottom panel), for each of four counties: Aberdeen, Clywd, Cumbria and Devon, in simulated outbreaks of FMD. Each set of simulations was run for a different method of deriving the underlying farm data: In (a) CSM, mean cow and sheep numbers on farms of that category, EQU, equal numbers of cows and sheep on every farm, RAN, spatially randomized locations, SHU, farms shuffled between locations, and TRU, the recorded data (see materials and methods for details). In (b) the different treatments are for derived land covers LC1–LC4, as described in the materials and methods section, using coarse and fine resolution land cover classification to describe potential farm land. The boxes represent the range of mean values for each of the 100 datasets used to run the model, and the whiskers show the 95% Prediction Interval (PI) for 1000 runs on each of the 100 generated datasets. NB: the methods TRU and EQU have no range of mean values.

We ran 1000 simulations for each data set in all four of our UK counties and investigated the epidemic size and duration as predicted by each data set. Should an epidemic occur in Cumbria, then an average of 1012 farms would become infected and an average of 1332 dangerous contacts (DCs) would be culled (figure 1a, top and middle panels). The mean epidemic duration according to the model is predicted to be 224 days (figure 1a, bottom panel). If farm locations are shuffled, the model performs well at predicting the mean total number of infected farms (1040), the total number of DCs (1365) and the epidemic duration (219 days). The same result is found to be true for Devon, Aberdeenshire and Clwyd, with accurate predictions found when farm locations are shuffled (though epidemic duration is slightly overestimated in all three cases).

As further uncertainty is built into the data, the ability of the model to predict epidemiological behaviour deteriorates. For the data sets “Shuffle CSM”, the model performs well in Aberdeenshire where epidemics are typically small, but slightly over predicts epidemic size and duration in Cumbria and Clwyd, whilst slightly under predicting these same quantities in Devon (figure 1a; see Table S1 for mean and 95% confidence intervals for all counties). If all farm sizes are assumed equal, the model is found to over predict the mean number of infected premises (IPs) and DCs in Cumbria and Clwyd by around 50% and marginally over predict epidemic duration, whilst significantly under predicting the same quantities in Devon. Whilst clustering of farms is preserved in these data sets, the lack of farm size heterogeneity appears to have a significant influence upon the ability of the model to accurately predict epidemic dynamics. If the true clustering is removed and farms are located randomly within each county, the model predicts small epidemics with a mean size of 90 infected farms in Cumbria, as compared to 1,012, lasting an average of 106 days, rather than 224. Similar under predictions occur for epidemic sizes and durations in Devon, Clwyd and Aberdeenshire (figure 1a). We conclude from this that, should precise farm locations in a region be unknown, a random location model is not able to make accurate predictions regarding epidemic size and duration.

Given that a random location model is a poor predictor of epidemic sizes and durations on the recorded farm location data, we now investigate epidemics simulated on the four land cover data set groups discussed above. Land Cover 1 (LC1) and Land Cover 2 (LC2) use aggregate classes to determine farm locations and figure 1b shows that the model significantly under predicts the number of IPs, DCs and epidemic duration in the counties of Clwyd and Cumbria for both of these data types. The model performs somewhat better in Devon and Aberdeenshire, though actually slightly over predicts epidemic size and duration in Devon and slightly under predicts these same quantities in Aberdeenshire. It appears that the AC data sets (LC1 and LC2), whilst proving better predictors than random location models, still differ significantly from epidemics simulated on the recorded farm location data.

Using the SC data, LC3, in which farms are located solely within “Improved grassland” and “Neutral grass” regions, predicted an average of 350 IPs in Devon and 32 in Aberdeenshire, compared with means of 330 and 31 respectively on the recorded farm location data. In Cumbria and Clwyd however, the mean number of IPs are found to be 818 and 214, slightly lower than the 1012 and 308 respectively on the recorded farm location data. Finally, the data sets in LC4 perform better than the data sets using AC classes and prove good predictors of epidemic duration but under predict epidemic sizes for all four counties (figure 1b; Table S1).

In conclusion, the models using data sets in LC3 in particular prove a significantly better predictor of epidemic size and duration on the recorded farm location data when compared with random location and AC constrained models. However, the slight underpredicting for some counties indicates that some elements of local clustering are not captured when using subclasses to determine farm locations.

### Ring culling

**Figure 2:**
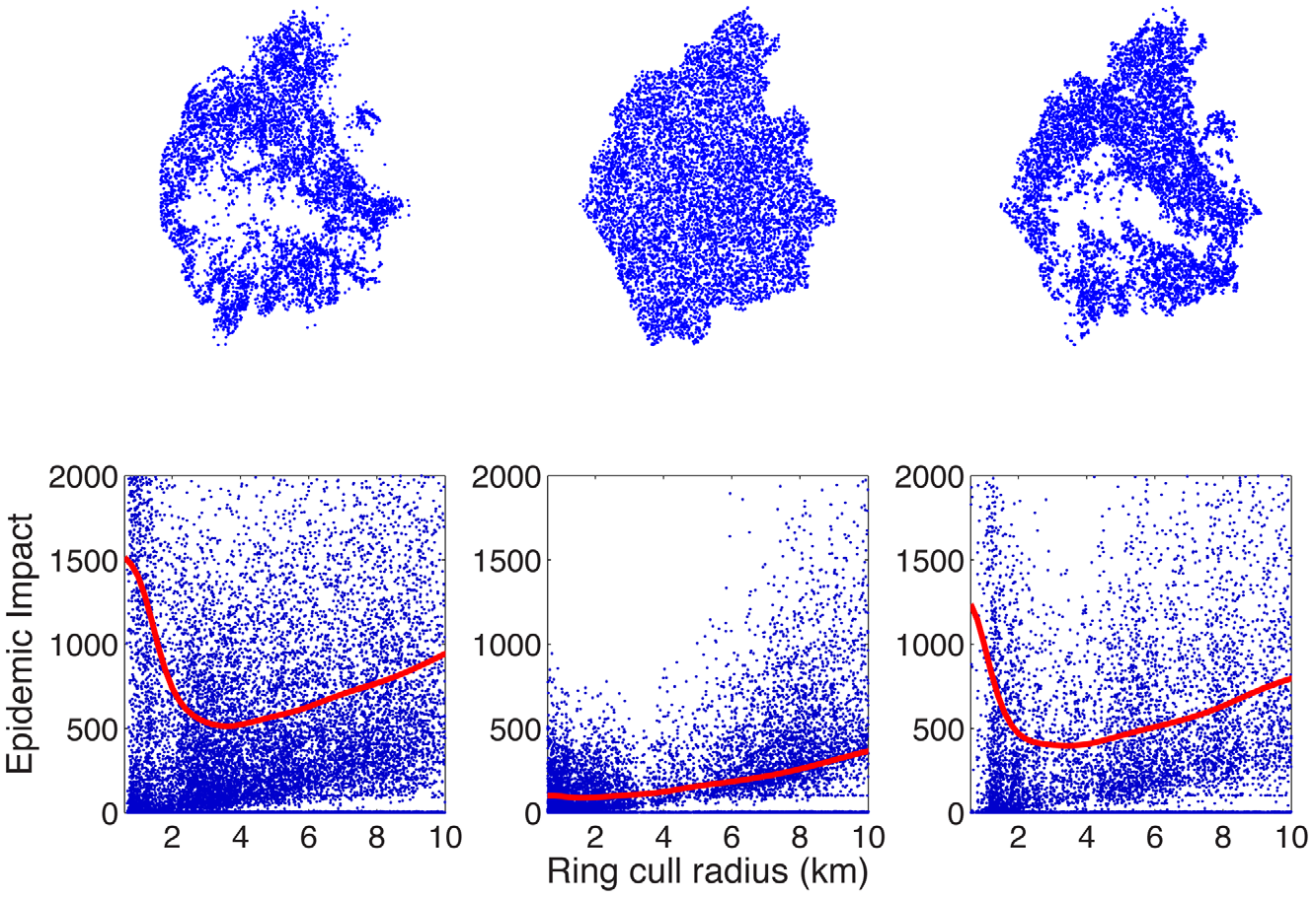
The upper panels show farm location data for Cumbria according to the agricultural census (left panel), one realisation assuming random locations (central panel) and one realisation assuming farms are located according to LC3 (right panel). The lower panels show the mean epidemic impact (solid line) and the raw outputs (black dots) against the radius of ring culling for the three data sets shown in the upper panels.

The model is now adapted to investigate the effect of the use of these synthetic farm databases upon preferred control strategies, in particular the optimal radius of a ring cull which minimises the Epidemic Impact, as summarised in table 2. Should epidemics be simulated on the recorded farm location data in Cumbria (figure 2, upper left panel), the optimal ring cull radius is found to be 3.6 km around all infected farms, resulting in a mean Epidemic Impact of 575 farms (figure 2, lower left panel). If precise farm sizes are known but coordinates are not known and farms are located randomly within Cumbria (figure 2, upper centre panel), the model predicts optimal ring cull radii of only 1.0 km to 2.2 km. An example for one of the random location data sets is shown in figure 2, central panels. If optimal ring cull radii as predicted by the random location models were applied to the recorded farm location data, the maximum potential increase in Epidemic Impact is 1398 farms. This implies that, for Cumbria, at least some knowledge of farm locations is required. In fact, in all four counties the ring cull radii predicted as optimal by the “Random” data sets would result in significant increases to the Epidemic Impact when applied to the recorded farm location data (table 2). Of course, in the event of an epidemic, it may be possible to use random location models parameterised to fit early outbreak data to provide policy advice, as investigated in previous work [17].

**Table 2:** Table showing the optimal ring cull radius *R_RC_* (in km) for epidemics simulated on the recorded farm location data and the generated data sets “Random”, LC1, LC2, LC3 and LC4 for Cumbria, Devon, Aberdeenshire and Clwyd (with the number of farms and size of the county).

County		RD	Random	Land Cover 1	Land Cover 2	Land Cover 3	Land Cover 4
Cumbria	*R_RC_*	3.6	1.6	2.8	2.8	3.5	3.5
(8035 farms; 6768 km^2^)	*R_RC(min)_-R_RC(max)_*		1.0–2.2	2.6–3.0	2.4–3.2	3.2–3.8	3.0–3.8
	*EI*	575	81	287	149	512	423
	*El_min_-El_max_*		71–102	264–308	136–162	445–561	386–439
	Δ*El_max_*		1398	199	305	17	30
Devon	*R_RC_*	2.8	1.2	2.0	1.8	2.8	2.9
(11177 farms; 6707 km^2^)	*R_RC(min)_-R_RC(max)_*		0.5–2.0	1.4–2.6	1.6–2.0	2.6–3.0	2.6–3.2
	*EI*	302	72	158	167	255	171
	*El_min_-El_max_*		65–85	139–179	153–189	243–267	160–186
	Δ*El_max_*		595	377	181	6	6
Aber'shire	*R_RC_*	2.4	0.3	0.1	0.3	2.4	2.2
(3086 farms; 6313 km^2^)	*R_RC(min)_-R_RC(max)_*		0.0–0.6	0.0–0.4	0.0–0.6	2.2–2.6	2.0–2.4
	*EI*	41	24	21	26	56	40
	*El_min_-El_max_*		6–35	8–38	12–41	52–63	36–45
	Δ*El_max_*		34	34	34	2	4
Clwyd	*R_RC_*	3.6	0.8	1.3	1.4	3.5	3.1
(3564 farms; 2910 km^2^)	*R_RC(min)_-R_RC(max)_*		0.2–1.8	0.8–1.8	0.8–1.8	3.2–3.8	2.8–3.4
	*EI*	369	154	162	165	289	182
	*EI_min_-EI_max_*		69–222	71–234	81–213	258–331	156–221
	Δ*EI_max_*		281	221	221	14	69

The optimal ring cull radius is defined to be the radius which minimises the mean Epidemic Impact (*EI*). The mean Epidemic Impact (*EI*) is listed for the recorded farm location data (RD), whilst for the simulated data sets, the mean (averaged over all 100 data sets) ring cull radius *R_RC_* (in km) and the mean Epidemic Impact (*EI*) and the range of values of optimal ring cull radii (*R_RC(min)_*-*R_RC(max)_*) and Epidemic Impacts (*EI_min_-EI_max_*) across each group of 100 data sets are shown. The maximum increase in epidemic impact, Δ*EI_max_*, if the optimal ring cull radius as determined on each of the 100 generated data sets in each group was carried out on the recorded farm location data for that county (as opposed to a ring cull at optimal radius for the recorded farm location data) is quoted in brackets. The number of farms and area of each county (in square kilometers) are also shown.

The models using LC1 and LC2 data sets under predict the optimal ring cull radius in all four counties resulting in a potential increase in Epidemic Impact of several hundred farms in Cumbria, Clwyd and Devon if these policies were applied to the recorded farm location data (table 2). In Aberdeenshire, where epidemics are typically small, the models predict that very low radius or no ring culling is optimal. Applying such a policy to the true data could, in a worst case scenario, almost double the mean Epidemic Impact. If SC models are used however, a much closer match to the optimal ring cull radius on the recorded farm location data is found for all four counties. LC3 appears the best predictor of optimal ring cull radius, with radii of 3.2 km–3.8 km predicted for Cumbria and Clwyd (c.f. 3.6 km for the recorded farm location data; see figure 2 right panels for an example data set), 2.6 km–3.0 km for Devon (c.f. 2.8 km for the recorded farm location data) and 2.2 km–2.6 km for Aberdeenshire (c.f. 2.4 km for the recorded farm location data). If these ring culls were applied to the recorded farm location data, the Epidemic Impact would increase by a maximum of 17, 6, 2 and 14 farms for Cumbria, Devon, Aberdeenshire and Clwyd respectively, compared with ring culling at the optimal radius. When LC4 is used, the model slightly underpredicts the optimal ring cull radius for some data sets in Cumbria and Clwyd, resulting in larger potential increases in Epidemic Impact of 30 and 69 farms respectively over ring culling at the optimal value. Our conclusion is therefore that, if the goal is to minimise farms lost to culling, the use of subclasses to predominantly determine cattle farm locations (as in LC3) provides the most robust representation of the recorded farm location data. However, all land cover informed data sets considered performed markedly better than models using random farm locations.

### Vaccination

Whilst ring culling may be considered as an additional control strategy to standard culling of IPs and DCs in the event of future epidemics in the UK or elsewhere, vaccination is a viable alternative to ring culling and is now part of DEFRA's contingency plan for FMD.

We therefore calculated the optimal vaccination radius that should be employed around all infected farms to minimize the Epidemic Impact. In Cumbria, vaccination at 34.2 km around all infected farms is found to minimize Epidemic Impact with a mean of 122 farms affected (table 3). In general, large vaccination radii around all infected farms are found to be optimal when an FMD outbreak is concentrated in a single region. Vaccination rings of 18.2 km, 30.4 km and 24.6 km are found to be optimal in Devon, Aberdeenshire and Clwyd respectively, when epidemics are simulated on the recorded farm location data (table 3). If farm locations are random, the model predicts optimal vaccination radii of between 17.0 km and 76.4 km and a potential increase in Epidemic Impact of 46 farms over the optimal strategy. Similar results are observed for Devon, Aberdeenshire and Clwyd, with the optimal strategy according to the random location databases resulting in increased Epidemic Impacts when applied to the recorded farm location data.

**Table 3:** Table showing the optimal vaccination radius *R_V_* (in km) for epidemics simulated on the recorded farm location data (RD) and the generated data sets “Random”, LC1, LC2, LC3 and LC4 for Cumbria, Devon, Aberdeenshire and Clwyd.

County		RD	Random	Land Cover 1	Land Cover 2	Land Cover 3	Land Cover 4
Cumbria	*R_V_*	34.2	42.0	44.6	41.5	38.9	36.6
	*R_V(min)_-R_V(max)_*		17.0–76.0	16.6–76.6	15.4–72.2	18.8–61.0	14.6–58.8
	*EI*	122	22	41	34	72	41
	*EI_min_-EI_max_*		19–25	22–65	18–61	35–97	31–54
	Δ*EI_max_*		46	46	51	25	23
Devon	*R_V_*	18.2	31.5	29.9	30.4	25.2	28.0
	*R_V(min)_-R_V(max)_*		10.2–71.0	11.6–63.4	14.8–65.4	16.0–48.0	14.2–52.2
	*EI*	70	21	37	38	33	29
	*EI_min_-EI_max_*		18–26	30–45	30–46	21–48	15–39
	Δ*EI_max_*		54	48	46	14	21
Aber'shire	*R_V_*	30.4	34.0	28.3	26.7	25.2	27.4
	*R_V(min)_-R_V(max)_*		12.4–65.4	10.2–52.4	12.2–50.0	16.0–41.6	15.4–45.4
	*EI*	31	9	10	10	17	15
	*EI_min_-EI_max_*		7–10	7–12	8–12	10–22	11–19
	Δ*EI_max_*		12	10	8	5	6
Clwyd	*R_V_*	24.6	42.8	35.5	32.4	28.2	27.5
	*R_V(min)_-R_V(max)_*		10–2–78.2	12.2–51.0	13.6–49.6	15.4–42.6	15.0–44.8
	*EI*	75	15	15	14	30	22
	*EI_min_-EI_max_*		12–19	13–16	12–17	21–40	17–28
	Δ*EI_max_*		14	9	8	8	8

The rows in the table are the same as for table 2.

The use of Land Cover data to determine farm locations only improves upon the random location model when SCs are used to ascertain optimal vaccination strategies. In the worst case scenario, the optimal vaccination radius according to the SC LC3 model would result in an increase in Epidemic Impact of 25, 14, 5 and 8 farms in Cumbria, Devon, Aberdeenshire and Clwyd respectively, compared with 46, 48, 10 and 9 farms respectively when the AC LC1 model is used. This supports the conclusions for ring culling, that land cover is able to dramatically improve the ability of the model to make epidemiological predictions when resolved at a sufficiently fine level of classification.

**Figure 3:**
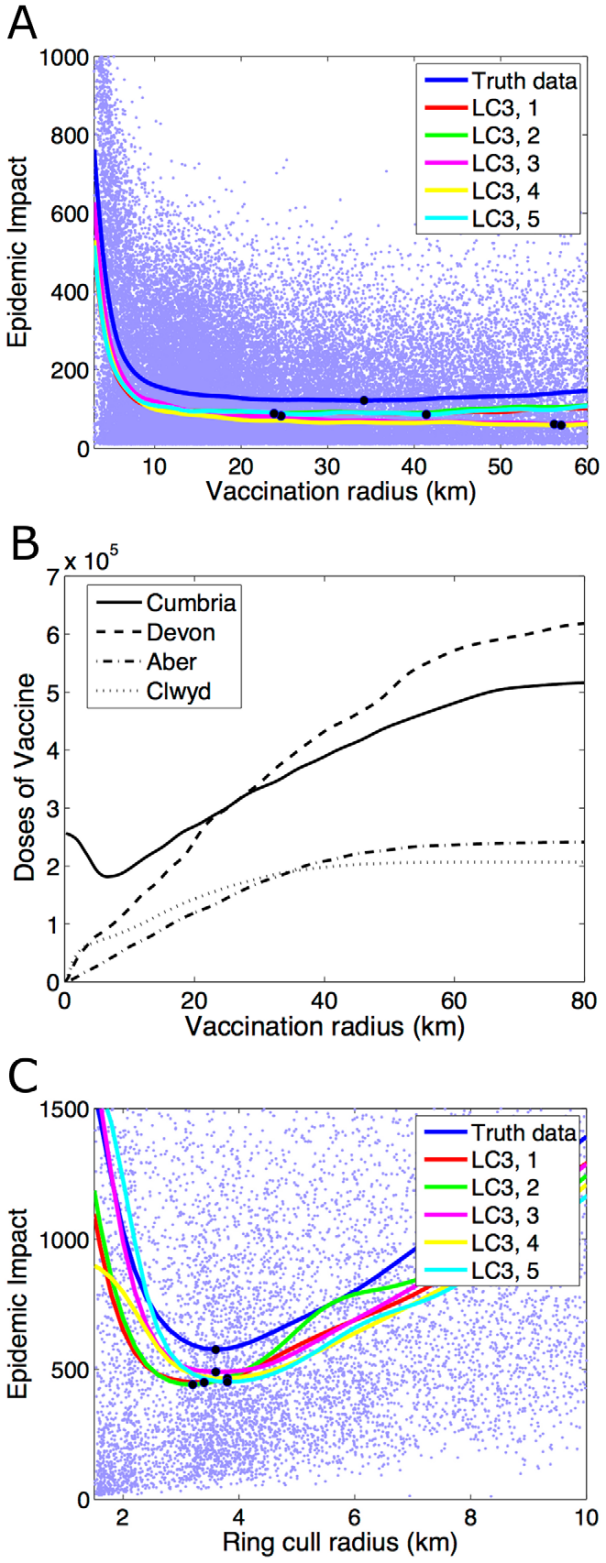
Graphs showing the mean Epidemic Impact against the radius of ring culling/vaccination for Cumbria only, for the recorded farm location data (blue line) and for five randomly selected data sets from LC3 for (a) ring culling and (b) vaccination. The results for the randomly selected data sets in each category are given by the red, green, yellow, magenta and cyan lines. The raw outputs for the recorded farm location data are shown by the blue dots. The black circles on each line indicate the radius which minimises the Epidemic Impact in each case. Each line is the mean of 20,000 model simulations and epidemics are seeded in Cumbria. (c) The number of doses of vaccine used as the vaccination radius varies for Cumbria (solid line), Devon (dashed line), Aberdeenshire (dash-dot line) and Clwyd (dotted line).

For outbreaks concentrated in single, contiguous regions, an analysis of the relationship between Epidemic Impact and ring cull radius reveals that the optimal radius is well defined, with a clear minimum in the curve (see figure 3a for LC3 for Cumbria). However, this does not hold true for vaccination. The optimal vaccination radius is generally high (around 20 km–40 km) and therefore vaccination rings tend to overlap. This results in a flattening out of the Epidemic Impact curve when the vaccination radius exceeds a certain critical value (see figure 3b for LC3 for Cumbria). Whilst vaccination of 34.2 km around all infected farms may in fact be optimal for Cumbria, the increase in Epidemic Impact is minimal when vaccination radii are between around 20 km and 40 km.

In all vaccination studies we have carried out to date, we made the assumption that the vaccination strategy is chosen to minimize the total number of farms with livestock culled, with no cost attached to the administration of the vaccination program. However, in a situation where the optimal strategy is not clearly defined, it may be important to select from a range of “acceptable” vaccination radii based upon the minimum number of doses of vaccine that would be used. When vaccination radii are small, vaccination is unable to control the epidemic in Cumbria and a huge number of doses are ultimately used as the number of farms infected increases dramatically. Whilst vaccination at 6 km minimizes the number of doses used in Cumbria (figure 3c), 6 km vaccination results in mean Epidemic Impacts around 3 times higher than when vaccination occurs at 20 km. Therefore, whilst 34.2 km is optimal in terms of minimizing the number of farms with livestock lost, a “better” strategy may be to vaccinate at 20 km, resulting in both a small epidemic and a reduced number of doses of vaccine used. Ultimately, a full economic cost analysis would allow for a rigorous identification of optimal strategies, taking into account the full cost of culling of all species of livestock, the cost of creating the vaccine and administering the vaccination campaign, as well as the cost associated with the introduction of movement restrictions and an international export ban.

### Data utility

The epidemiological analysis presented in this paper concludes that land cover data can be useful for models of control predictions for foot-and-mouth disease spread. However, we found that for the Land Cover Map 2000 for the UK, this only generates accurate results when subclass (SC) data are used to inform farm locations. It is therefore important to investigate what precisely is being captured by the subclass data that is being missed in less resolved data.

**Figure 4:**
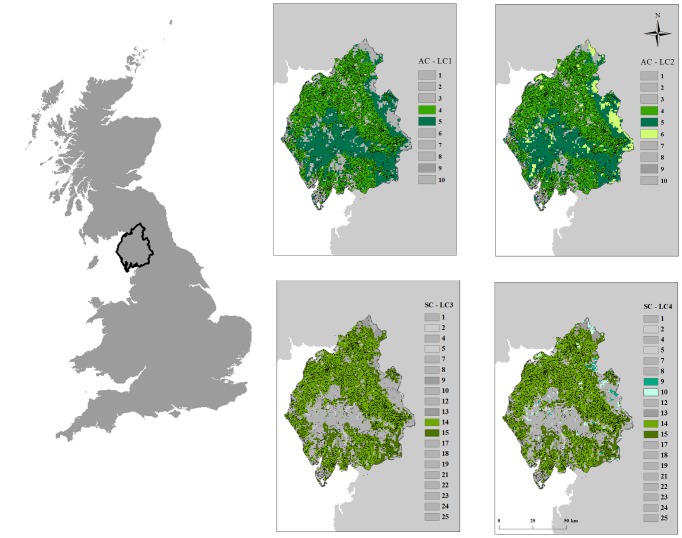
Land cover parcels for the 10 aggregate classes (top two panels) and 25 subclasses (bottom two panels) according to Land Cover Map 2000. Land cover classes that are assumed to host livestock farms are colored in shades of green, whilst all other land cover classes are colored in shades of grey according to LC1 (top left panel), LC2 (top right panel), LC3 (bottom right panel) and LC4 (bottom left panel). Figure provided courtesy of the Centre for Ecology and Hydrology (NERC (CEH)).

When examining the recorded farm location data, we see that the highest density of farms are found in the North and East of Cumbria, with few farms in the centre of the County (figure 2, upper left panel). This spatial heterogeneity is not captured either when random locations are assumed (figure 2, upper centre panel), or when ACs are used to determine farm locations. However, when SCs are used, the generated spatial data are found to closely correspond with the recorded farm location data (figure 2, upper right panel). In particular the Lake District, in the centre of Cumbria where no farms are located, is clearly visible when SCs are used to determine farm locations. In our initial analysis, it was decided that aggregate classes 4 and 5, “Improved Grassland” and “Semi-natural grass” respectively, were most likely to be correlated with cattle farm locations. However, when we compare this to spatial data from Land Cover Map for Cumbria, we see that the Lake District, where few farms are located, is predominantly in the “Semi-natural grass” category (figure 4, top panels). Hence the use of ACs would preferentially locate farms in that region and hence not capture the spatial clustering evident within Cumbria. SC data proves much more useful in determining farm locations, with SCs 14 and 15 closely mirroring true farm locations, whilst SC 20 (Fen, Marsh and Swamp) appears to account for the majority of the Lake District (figure 4, bottom panels).

Whilst not quite as marked, a similar pattern is found in Devon. From the recorded farm location data (Figure S1, left panel) we see that there are very few farms in a large proportion of the South West of the county. This region is dominated by Dartmoor National Park. If we assume random locations for farms, the resultant density map bears little resemblance to the recorded data (Figure S1, centre panel). However, when the LC3 data set is used, a much better approximation to the recorded data is observed, with the absence of farms in the Dartmoor region clearly visible (Figure S1, right panel).

We now examine how well our synthetic data capture the spatial clustering of farms observed in the recorded farm location data. A simple method to estimate the statistical difference between the recorded and synthetic data sets is to calculate the root mean square error (RM) between actual farm locations (as determined by the agricultural census) and predicted farm locations (as predicted by our LC data sets described above), averaged over all 100 realisations of the given synthetic data set. In order to calculate the value of RM, we project a uniform set of square grids of a given size over each county and calculate the RM over all grids as the size of the grids vary. Therefore:
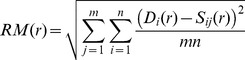
where *n* is the total number of grids of length *r*, *D_i_(r)* is the number of farms in grid *i* in the recorded data, *S_ij_(r)* is the number of farms in grid *i* according to synthetic data set *j*, and *m* is the total number of synthetic data sets generated (set to 100 in this paper).

**Figure 5:**
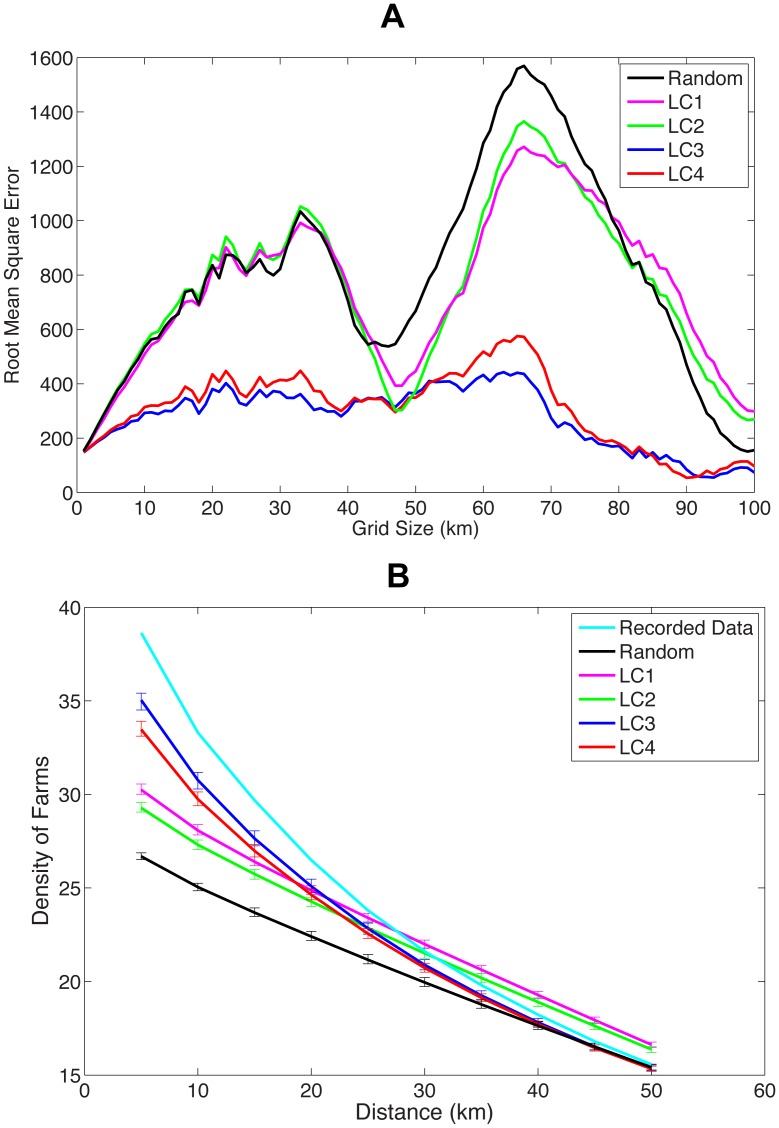
Graphs showing (a) the root mean square error (RM) between the synthetic data and the recorded data for Cumbria as the radius is varied and (b) the mean density of farms (per km^2^) within a given radius of all other farms, averaged over all realisations of each synthetic data set. 95% confidence intervals are also shown for each data set. In both (a) and (b) results are shown for the random data (black line), LC1 (magenta line), LC2 (green line), LC3 (blue line) and LC4 (red line). In (b), spatial clustering for the recorded data is indicated by the cyan line.

This metric is determined for the random location data sets and LC1–LC4 as the length *r* of each grid varies from 1 km to 100 km. The results are summarised in figure 5 for Cumbria. For low values of *r*, the majority of grid squares have few farms in both the recorded and synthetic data and therefore the value of RM is low for all synthetic data sets. However, as *r* is increased, the spatial error in the synthetic data becomes evident as RM increases (see figure 5a for Cumbria). The LC1 and LC2 data sets are found to perform no better than the random data sets, whilst the root mean square error is found to be lowest when LC3 data sets are used. In Cumbria, as *r* increases beyond approximately 30 km, the value of RM decreases for all data sets, before increasing once again for *r* greater than approximately 50 km. This unusual phenomenon may be because of the demographic nature of Cumbria – the presence of the Lake District in the centre of the county, correlated with an absence of farms, may indicate that there is little clustering present in the data at length scales of between 30 and 50 km and hence root mean square errors over grid scales of this range of sizes would decrease. As *r* is increased yet further, the value of RM increases once again before ultimately decreasing to zero at the point that the whole of Cumbria is confined within a single grid square. We find similar, but less marked behaviour in Devon, owing to the presence of Dartmoor in the South West of the County. We conclude that, whilst clustering at all scales is not captured precisely using land cover, the data sets using subclasses perform significantly better than those using more coarse scale aggregate classes.

As an alternate measure of the clustering of the synthetic data sets, we calculate the density of farms (per km^2^) within a given radius *r* of all farms and average this over all farms and all replicates of each synthetic data set. We can then compare all land cover and the random location data sets to the recorded data set and determine how well the synthetic data sets capture both short and long range clustering. Results are summarised in figure 5b for Cumbria. All data sets appear to underestimate local clustering in Cumbria. This is unsurprising – even subclass data will not be able to capture the fine-scale local clustering behavior of farm locations. However, local clustering is found to be much higher in the LC3 and LC4 data sets than in the LC1, LC2 and random location data sets. As the radius *r* increases, the mean density of farms in the recorded data is more accurately captured in all data sets. We note that synthetic data sets LC1 and LC2 have higher clustering than the recorded data at scales greater than around 30 km. The absence of farms in the Lake District will not be captured by these data sets and therefore we would expect increased clustering in these data sets for radii between around 30 and 50 km. A much closer fit to the spatial clustering present in the recorded data is found with data sets LC3 and LC4 (figure 5b, blue and red lines for LC3 and LC4 respectively). Similar behavior is found for all other counties analysed in this paper (see Figure S2). Whilst we are not capturing all the spatial clustering properties of the recorded data by using land cover data, it is apparent from this paper that the level of clustering in the synthetic data sets LC3 is sufficient to provide accurate predictions of epidemiological properties in the event of an outbreak of disease.

## Discussion

The ability of infectious disease models to make accurate epidemiological predictions – particularly for control - is highly dependent upon the accuracy of spatio-temporal epidemic and location data. In countries such as the USA, when farm data are only available at an aggregate scale, land use and other geographic data may be useful for creating surrogates for precise farm locations. The use of farm location data in the UK combined with Land Cover Map 2000 enables an investigation into the utility of land use maps to estimate farm locations and the effect of the estimation process upon size, duration and preferred control strategies for future epidemics.

It is clear that the loss of clustering which occurs when assuming random locations for farms causes a massive underestimate in epidemic sizes and durations, which could lead to inaccurate culling and vaccination recommendations to policy makers. The use of broadly defined landscape classes to determine farm locations improved this somewhat, but the increased Epidemic Impacts which would occur from adopting suggested vaccination or ring culling policies are still significant. However, when farm locations were correlated to more resolved landscape classes, in particular “Improved Grassland” and “Neutral grass”, the model captured the spatio-temporal epidemic profile well and provides accurate predictions for control. This proves an interesting result – whilst land use can act as an accurate predictor for farm locations, poorly resolved data could generate model predictions that deviate significantly from observations.

The land cover data set that performed best in this analysis, LC3, was chosen to correlate with cattle farm locations. LC4 was extended to accommodate farms located within shrub heath and montane habitats, to account for hill sheep farm locations. The model using LC4 data did not perform as well as LC3 and we conclude that this data set, which would inaccurately locate some large cattle farms in hill farm locations, would lose elements of clustering of large farms and so underestimate epidemic sizes and durations. The impact of underestimating epidemic sizes and durations on control could lead to significantly larger ‘escaped’ epidemics, and add loss of livestock and financial resources. In addition, this particular example may reveal an added conundrum, wherein we are over-estimating potential farm habitat and overshooting the information optimum, even with the finely resolved landscape classifications.

It is important to note that this paper only focuses on spread of infection *within* counties in the UK and does not address the issue of the influence of precise farm locations upon *between* county spread. In Cumbria, the presence of the Lake District in the center of the county would suggest that a random location model would predict lower livestock densities near county borders and therefore underestimate cross-border transmission. Equally, models of counties with natural boundaries at their borders (e.g. mountain ranges or lakes) would overestimate cross border transmission in a random location model. The use of land cover data of sufficient resolution would provide a better estimate of farm locations in close proximity to county boundaries and hence a more accurate prediction of cross-border spread of disease.

The advantage of this approach over previous work [17] is that it enables epidemiological predictions to be made in the advance of an epidemic, based upon known strain characteristics. It also provides a framework for assessing the potential for epidemic impact and spread in low data environments, and identifies the pitfalls and potential costs of too little information. An obvious extension of this work would be to utilise a Bayesian statistical inference approach [25]–[27] to fit models that utilise land cover data to historical epidemic data (e.g. the early stages of the 2001 FMD epidemic in the UK) and investigate the accuracy with which these models can predict the spatio-temporal dynamics of the epidemic and preferred intervention strategies. This would provide insights into the utility of such models to be used during the early stages of future epidemics to provide robust policy advice.

## Supporting Information

Dataset S1 R script designed to generate 100 sets of spatially random points within a non-continuous raster representing certain landcover types.
(TXT)

Dataset S2 R script designed to generate 100 sets of spatially random points within a single polygon.
(TXT)

Figure S1 Data for Devon showing the density of farms according to (a) the truth data, (b) assuming random locations (c) when locating farms using “Land Cover 3” data. Farm densities are shown in parcels of 4 square kilometers and the color scale shows the number of farms per square kilometer within each grid square.
(TIF)

Figure S2 Graphs showing the average density of farms against radius around each farm as the radius varies for Aberdeenshire (upper panel), Clwyd (middle panel) and Devon (lower panel). Each graph shows density for the recorded data (cyan line), random data (black line), “Land Cover 1” data (magenta line), “Land Cover 2” data (green line), “Land Cover 3” data (blue line) and “Land Cover 4” data (red line).
(TIF)

Figure S3 Graph showing the mean and 95% confidence intervals of the Epidemic Impact in Cumbria for the recorded data (black), the generated data set “Random” (red) and the land cover derived data set LC3 (blue) as the transmissibility of cattle (*T_c_*) is varied. The arrow indicates the value of *T_c_* that was used in the model to simulate the UK 2001 epidemic.
(TIF)

Table S1 The mean and 95% confidence intervals of number of IPs, DCs and the duration (in days) for epidemics seeded in Cumbria, Devon, Clwyd and Aberdeenshire. Epidemics are simulated using the recorded data, the generated data sets “Shuffle”, “Shuffle CSM”, “All Equal” and “Random” and the land cover derived data sets LC1–LC4.
(PDF)

Table S2 The proportion of pixels (1 km^2^) in each county in each land cover class, for all counties, aggregate class (AC) and subclass (SC) in the LCM 2000 database. Cover classes in LC1–LC4 are denoted in bold.
(PDF)

Table S3 Proportions (MEAN and SD) of farms occurring in each land cover class, for recorded data (aggregate class, AC, and subclass, SC), Random (AC, SC); cover classes in LC1–LC4 are in bold, and are summarized in the final table of the four Land cover scenarios in the paper - LC1–LC4.
(PDF)

Table S4 The mean and 95% confidence intervals of number of IPs, DCs for epidemics seeded in Cumbria with no ring culling, and the optimal ring cull radius *R_RC_* (in km) when ring culling is included, for epidemics on the recorded data, the generated data set “Random” and the land cover derived data set LC3. Epidemics are simulated using the UK 2001 dispersal kernel, a kernel with twice the height and half the width of the UK kernel (Kernel 2) and a kernel with half the height and twice the width of the UK kernel (Kernel 3). For the simulated data sets, the range of values of optimal ring cull radii (*R_RC(min)_*-*R_RC(max)_*) across each group of 100 data sets are shown.
(PDF)

Text S1 Supplementary information providing details of sensitivity analyses and describing supplementary figures S1 to S3 and supplementary tables S1 to S4.
(PDF)

## References

[1] Anderson I (2002). Foot and Mouth Disease 2001: Lessons to be Learned Enquiry. London: The Stationary Office.

[2] Keeling MJ, Woolhouse MEJ, Shaw DJ, Matthews L, Chase-Topping ME, et al (2001) Dynamics of the 2001 UK foot and mouth epidemic: stochastic dispersal in a heterogeneous landscape. Science 294: 813–817. 11679661 10.1126/science.1065973

[3] Ferguson NM, Donnelly CA, Anderson RM (2001a) Transmission intensity and impact of control policies on the foot and mouth epidemic in Great Britain. Nature 413: 542–548. 11586365 10.1038/35097116

[4] Tildesley MJ, Bessell PR, Keeling MJ, Woolhouse MEJ (2009) The role of pre-emptive culling in the control of Foot-and-Mouth Disease. Proc Roy Soc B 276: 3239–3248. 10.1098/rspb.2009.0427 PMC2817163 19570791

[5] Diggle PJ (2006) Spatio-temporal point processes, partial likelihood, foot and mouth disease. Stat Methods Med Res 25: 325–336. 10.1191/0962280206sm454oa 16886734

[6] Bessell PR, Shaw DJ, Savill NJ, Woolhouse MEJ (2009) Statistical modeling of holding level susceptibility to infection during the UK 2001 Foot and Mouth Disease epidemic. Int J Infect Dis 14: E210–E215. 19647465 10.1016/j.ijid.2009.05.003

[7] Deardon R, Brooks SP, Grenfell BT, Keeling MJ, Tildesley MJ, et al (2009) Inference for individual-level models of infectious diseases in large populations. Stat Sin 20: 239–261. PMC4578172 26405426

[8] Keeling MJ, Woolhouse MEJ, May RM, Davies G, Grenfell BT (2003) Modeling vaccination strategies against foot-and-mouth disease. Nature 421: 136–142. 12508120 10.1038/nature01343

[9] Tildesley MJ, Savill NJ, Shaw DJ, Deardon R, Brooks SP, et al (2006) Optimal reactive vaccination strategies for a foot-and-mouth outbreak in Great Britain. Nature 440: 83–86. 16511494 10.1038/nature04324

[10] Tildesley MJ, Keeling MJ (2008) Modeling foot-and-mouth disease: A comparison between the UK and Denmark. Prev Vet Med 85: 107–124. 18328581 10.1016/j.prevetmed.2008.01.008

[11] Ferguson NM, Cummings DAT, Cauchemez S, Fraser C, Riley S, et al (2005) Strategies for containing an emerging influenza pandemic in Southeast Asia. Nature 437: 209–214. 16079797 10.1038/nature04017

[12] Gudelj I, White KAJ (2004) Spatial heterogeneity, social structure and disease dynamics of animal populations. Theor Popul Biol 66: 139–149. 15302223 10.1016/j.tpb.2004.04.003

[13] Swinton J, Harwood J, Grenfell BT, Gilligan CA (1998) Persistence thresholds for phocine distemper virus infection in harbour seal Phoca vitulina metapopulations. J An Ecol 67: 54–68.

[14] Hugh-Jones ME (1972) Epidemiological studies on 1967–1968 Foot and Mouth Epidemic - attack rates and cattle density. Res Vet Sci 13: 411–417. 4635111

[15] Ferguson NM, Donnelly CA, Anderson RM (2001b) The foot-and-mouth epidemic in Great Britain: pattern of spread and impact of interventions. Science 292: 1155–1160. 11303090 10.1126/science.1061020

[16] Taylor NM, Honhold N, Paterson AD, Mansley LM (2004) Risk of foot-and-mouth disease associated with proximity in space and time to infected premises and the implications for control policy during the 2001 epidemic in Cumbria. Vet Rec 154: 617–626. 15180396 10.1136/vr.154.20.617

[17] Tildesley MJ, House TA, Bruhn MC, Curry RJ, O'Neil M, et al (2010) Impact of spatial clustering on disease transmission and optimal control. Proc Natl Acad Sci U S A 107: 1041–1046. 19955428 10.1073/pnas.0909047107 PMC2824282

[18] Dion E, VanSchalkwky L, Lambin EF (2010) The landscape epidemiology of foot-and-mouth disease in South Africa: A spatially explicit multi-agent simulation. Ecol Model 222: 2059–2072.

[19] Lambin EF, Tran A, Vanwambeke SO, Linard C, Soti V (2010) Pathogenic landscapes: interactions between land, people, disease vectors, and their animal hosts. Int J Health Geogr 9: 54. 20979609 10.1186/1476-072X-9-54 PMC2984574

[20] Ostfield RS, Glass GE, Keesing F (2005) Spatial epidemiology: an emerging (or re-emerging) discipline. Trends Ecol Evol 20: 328–336. 16701389 10.1016/j.tree.2005.03.009

[21] Rogers DJ, Randolph SE (1991) Mortality rates and population density of tsetse flies correlated with satellite imagery. Nature 351: 739–741. 2062367 10.1038/351739a0

[22] Estrada-Pena A (2002) Increasing habitat suitability in the United States for the tick that transmits Lyme disease: a remote sensing approach. Environ Health Perspect 110: 635–640. 12117639 10.1289/ehp.110-1240908 PMC1240908

[23] Fuller RM, Smith GM, Sanderson JM, Hill RA, Thomson AG (2002) The UK Land Cover Map 2000: Construction of a Parcel-Based Vector Map from Satellite Images. Cartogr J 39: 15–25.

[24] Fuller RM, Cox R, Clarke RT, Rothery P, Hill RA, et al (2005) The UK land cover map 2000: Planning, construction and calibration of a remotely sensed, user-oriented map of broad habitats. Int J Appl Earth Obs 7: 202–216.

[25] Diggle PJ (2006) Spatio-temporal point processes, partial likelihood, foot and mouth disease. Stat Methods Med Res 15: 325–336. 16886734 10.1191/0962280206sm454oa

[26] Jewell CP, Keeling MJ, Roberts GO (2008) Predicting undetected infections during the 2007 foot-and-mouth disease outbreak. J R Soc Interface 6: 1145–1151. 19091686 10.1098/rsif.2008.0433 PMC2817150

[27] King AA, Ionides EL, Pascual M, Bouma MJ (2008) Inapparent infections and cholera dynamics. Nature 454: 877–879. 18704085 10.1038/nature07084

