# Supplementary figures and images for: Disease Prevention versus Data Privacy: Using Landcover Maps to Inform Spatial Epidemic Models
Source: PLoS Comput Biol. 2012 Nov 1;8(11):e1002723. doi: 10.1371/journal.pcbi.1002723 (PMC3486837; doi:10.1371/journal.pcbi.1002723)

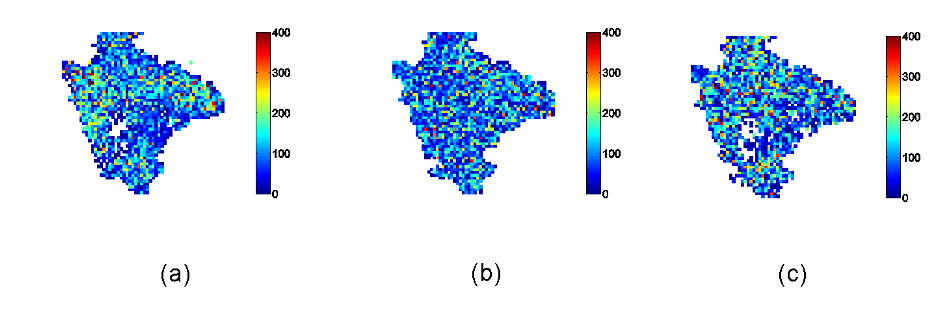

Supplement: Figure S1 — Data for Devon showing the density of farms according to (a) the truth data, (b) assuming random locations (c) when locating farms using “Land Cover 3” data. Farm densities are shown in parcels of 4 square kilometers and the color scale shows the number of farms per square kilometer within each grid square. (TIF) [file pcbi.1002723.s003.tif]

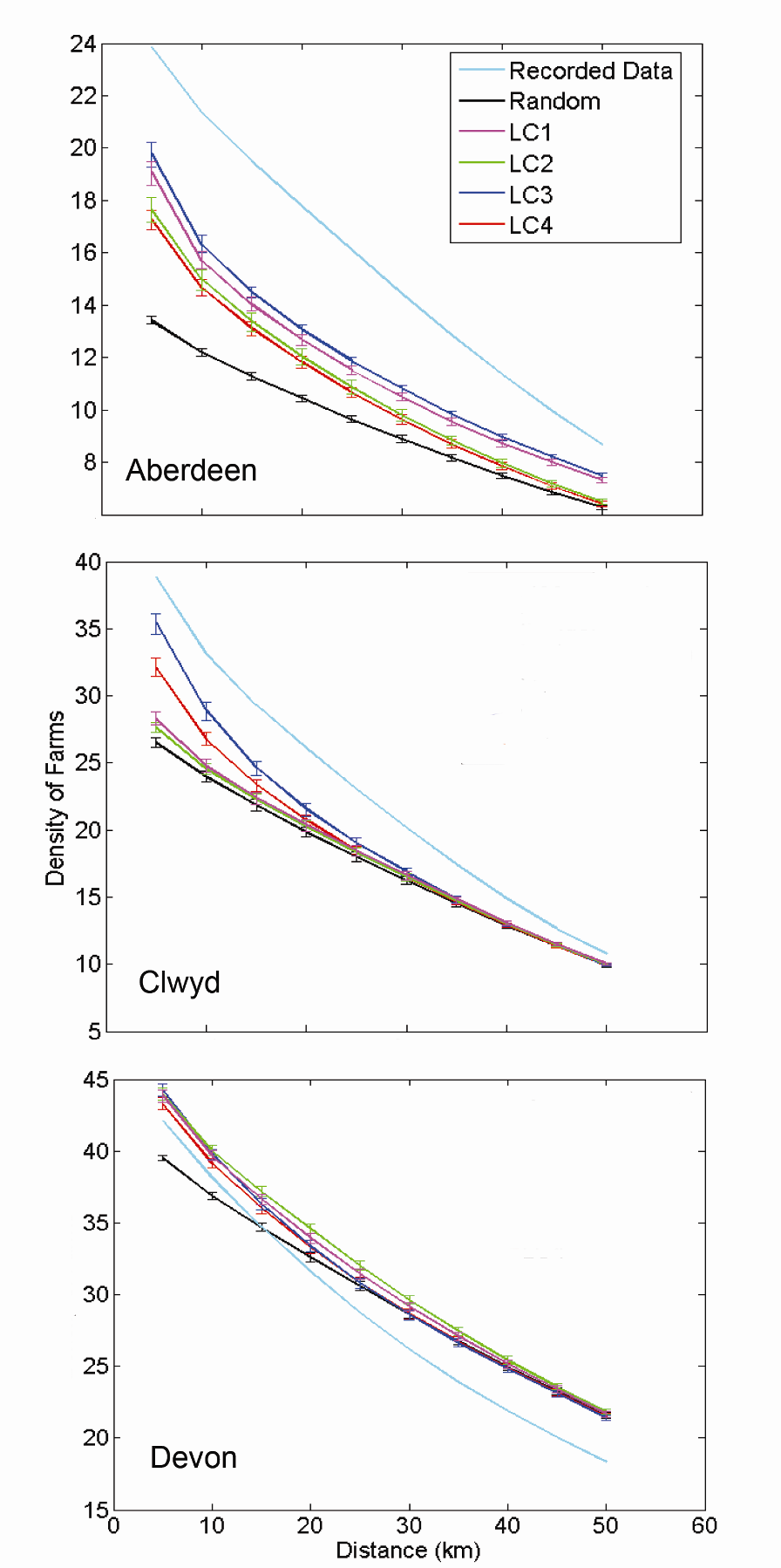

Supplement: Figure S2 — Graphs showing the average density of farms against radius around each farm as the radius varies for Aberdeenshire (upper panel), Clwyd (middle panel) and Devon (lower panel). Each graph shows density for the recorded data (cyan line), random data (black line), “Land Cover 1” data (magenta line), “Land Cover 2” data (green line), “Land Cover 3” data (blue line) and “Land Cover 4” data (red line). (TIF) [file pcbi.1002723.s004.tif]

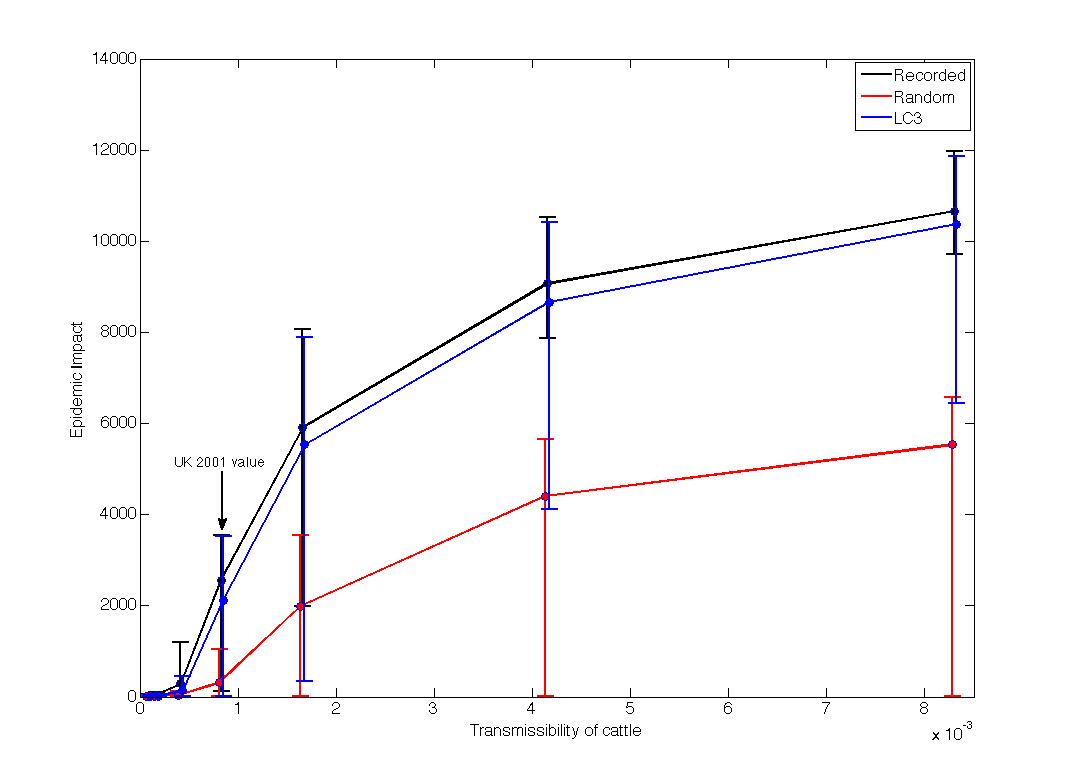

Supplement: Figure S3 — Graph showing the mean and 95% confidence intervals of the Epidemic Impact in Cumbria for the recorded data (black), the generated data set “Random” (red) and the land cover derived data set LC3 (blue) as the transmissibility of cattle (Tc) is varied. The arrow indicates the value of Tc that was used in the model to simulate the UK 2001 epidemic. (TIF) [file pcbi.1002723.s005.tif]
