# Supplementary material for: Disease Prevention versus Data Privacy: Using Landcover Maps to Inform Spatial Epidemic Models
Source: PLoS Comput Biol. 2012 Nov 1;8(11):e1002723. doi: 10.1371/journal.pcbi.1002723 (PMC3486837; doi:10.1371/journal.pcbi.1002723)
Supplement: Table S1 — The mean and 95% confidence intervals of number of IPs, DCs and the duration (in days) for epidemics seeded in Cumbria, Devon, Clwyd and Aberdeenshire. Epidemics are simulated using the recorded data, the generated data sets “Shuffle”, “Shuffle CSM”, “All Equal” and “Random” and the land cover derived data sets LC1–LC4. (PDF) [file pcbi.1002723.s006.pdf]

Supplementary Table 1

| | | Cumbria | Devon | Clwyd | Aberdeenshire |
|---|---|---|---|---|---|
| Recorded Data | IPs | 1012 (42-1280) | 150 (6-593) | 308 (7-548) | 32 (6-71) |
| | DCs | 1332 (62-1706) | 330 (9-1350) | 392 (5-663) | 31 (3-73) |
| | Duration | 224 (39-356) | 118 (30-305) | 159 (36-221) | 61 (30-133) |
| SHU | IPs | 1040 (10-1486) | 149 (7-532) | 322 (8-621) | 34 (6-165) |
| | DCs | 1365 (11-1986) | 329 (11-1178) | 403 (7-698) | 39 (3-187) |
| | Duration | 217 (41-331) | 128 (32-314) | 166 (33-308) | 73 (29-170) |
| CSM | IPs | 1094 (10-1497) | 115 (6-312) | 335 (7-649) | 33 (6-134) |
| | DCs | 1366 (10-1951) | 295 (10-969) | 412 (7-760) | 34 (3-147) |
| | Duration | 228 (41-380) | 112 (33-265) | 178 (34-354) | 67 (29-164) |
| EQU | IPs | 1570 (9-2109) | 62 (6-269) | 472 (7-807) | 33 (6-139) |
| | DCs | 2060 (111-2693) | 127 (10-554) | 577 (6-933) | 34 (3-148) |
| | Duration | 243 (39-412) | 96 (31-246) | 207 (35-387) | 69 (30-268) |
| RAN | IPs | 90 (6-377) | 56 (6-216) | 31 (6-104) | 9 (6-19) |
| | DCs | 106 (5-446) | 112 (9-433) | 32 (4-105) | 5 (0-13) |
| | Duration | 106 (31-275) | 84 (31-190) | 63 (29-141) | 38 (25-59) |
| LC1 | IPs | 324 (7-861) | 160 (7-571) | 50 (6-179) | 26 (6-76) |
| | DCs | 284 (8-883) | 406 (12-1327) | 56 (5-204) | 27 (3-83) |
| | Duration | 192 (33-385) | 133 (33-324) | 77 (30-179) | 59 (29-117) |
| LC2 | IPs | 228 (7-724) | 184 (7-615) | 43 (6-154) | 21 (6-65) |
| | DCs | 254 (9-765) | 295 (10-969) | 47 (4-169) | 20 (2-68) |
| | Duration | 228 (41-380) | 141 (34-339) | 72 (30-163) | 55 (28-110) |
| LC3 | IPs | 818 (8-1286) | 159 (7-576) | 214 (7-525) | 30 (6-93) |
| | DCs | 1062 (9-1622) | 350 (11-1246) | 367 (6-639) | 32 (3-103) |
| | Duration | 236 (37-428) | 132 (33-327) | 141 (33-294) | 62 (29-129) |
| LC4 | IPs | 682 (7-1187) | 132 (6-496) | 112 (6-365) | 29 (6-61) |
| | DCs | 881 (8-1482) | 287 (11-1072) | 134 (5-434) | 28 (2-60) |
| | Duration | 231 (37-432) | 121 (32-304) | 127 (31-248) | 58 (28-102) |
