# Supplementary material for: Disease Prevention versus Data Privacy: Using Landcover Maps to Inform Spatial Epidemic Models
Source: PLoS Comput Biol. 2012 Nov 1;8(11):e1002723. doi: 10.1371/journal.pcbi.1002723 (PMC3486837; doi:10.1371/journal.pcbi.1002723)
Supplement: Table S2 — The proportion of pixels (1 km2) in each county in each land cover class, for all counties, aggregate class (AC) and subclass (SC) in the LCM 2000 database. Cover classes in LC1–LC4 are denoted in bold. (PDF) [file pcbi.1002723.s007.pdf]

AC	*Aberdeen*	*Clwyd*	*Cumbria*	*Devon*	*All*
1	0.01	0.02	0.02	0.04	0.02
2	0.10	0.05	0.03	0.01	0.05
3	0.42	0.02	0.04	0.21	0.20
**4**	**0.15**	**0.41**	**0.48**	**0.60**	**0.41**
**5**	**0.04**	**0.38**	**0.33**	**0.07**	**0.18**
**6**	**0.25**	**0.06**	**0.06**	**0.03**	**0.11**
7	0.02	0.04	0.01	0.03	0.02
8	0.00	0.00	0.01	0.00	0.00
9	0.00	0.01	0.01	0.00	0.01
10	0.01	0.00	0.00	0.01	0.00

SC	*Aberdeen*	*Clwyd*	*Cumbria*	*Devon*	*All*
1	0.01	0.00	0.00	0.01	0.01
2	0.00	0.00	0.01	0.00	0.00
3	0.00	0.00	0.00	0.00	0.00
4	0.00	0.00	0.01	0.00	0.00
5	0.00	0.00	0.00	0.00	0.00
6	0.00	0.00	0.00	0.00	0.00
7	0.00	0.00	0.00	0.00	0.00
**8**	**0.01**	**0.00**	**0.03**	**0.01**	**0.01**
**9**	**0.14**	**0.04**	**0.01**	**0.00**	**0.05**
**10**	**0.02**	**0.01**	**0.02**	**0.01**	**0.02**
**11**	**0.07**	**0.00**	**0.00**	**0.00**	**0.02**
12	0.01	0.03	0.02	0.05	0.03
13	0.11	0.06	0.03	0.01	0.05
**14**	**0.17**	**0.43**	**0.52**	**0.68**	**0.45**
**15**	**0.02**	**0.07**	**0.10**	**0.01**	**0.05**
**16**	**0.00**	**0.00**	**0.00**	**0.00**	**0.00**
**17**	**0.00**	**0.00**	**0.06**	**0.00**	**0.02**
**18**	**0.00**	**0.07**	**0.03**	**0.01**	**0.02**
**19**	**0.01**	**0.23**	**0.11**	**0.05**	**0.08**
**20**	**0.00**	**0.00**	**0.00**	**0.00**	**0.00**
21	0.00	0.01	0.00	0.08	0.03
22	0.40	0.01	0.02	0.04	0.13
23	0.00	0.00	0.00	0.00	0.00
24	0.02	0.03	0.01	0.02	0.02
25	0.00	0.01	0.00	0.00	0.00
