# Supplementary material for: Disease Prevention versus Data Privacy: Using Landcover Maps to Inform Spatial Epidemic Models
Source: PLoS Comput Biol. 2012 Nov 1;8(11):e1002723. doi: 10.1371/journal.pcbi.1002723 (PMC3486837; doi:10.1371/journal.pcbi.1002723)
Supplement: Table S3 — Proportions (MEAN and SD) of farms occurring in each land cover class, for recorded data (aggregate class, AC, and subclass, SC), Random (AC, SC); cover classes in LC1–LC4 are in bold, and are summarized in the final table of the four Land cover scenarios in the paper - LC1–LC4. (PDF) [file pcbi.1002723.s008.pdf]

Aberdeen

Recorded				Random					
AC		SC		AC	MEAN	SD	SC	MEAN	SD
1	0.01	1	0.00	1	0.01	0.00	1	0.01	0.00
2	0.04	7	0.00	2	0.10	0.01	2	0.00	0.00
3	0.60	**8**	**0.00**	3	0.42	0.01	7	0.00	0.00
**4**	**0.28**	**9**	**0.01**	**4**	**0.15**	**0.01**	**8**	**0.01**	**0.02**
**5**	**0.03**	**10**	**0.00**	**5**	**0.04**	**0.00**	**9**	**0.13**	**0.02**
**6**	**0.03**	**11**	**0.00**	**6**	**0.25**	**0.01**	**10**	**0.02**	**0.01**
7	0.00	12	0.01	7	0.02	0.00	**11**	**0.07**	**0.01**
9	0.00	13	0.05	8	0.00	0.00	12	0.01	0.02
10	0.00	**14**	**0.32**	9	0.00	0.00	13	0.11	0.01
		**15**	**0.02**	10	0.01	0.00	**14**	**0.17**	**0.03**
		**18**	**0.00**				**15**	**0.02**	**0.00**
		**19**	**0.01**				**18**	**0.00**	**0.01**
		22	0.57				**19**	**0.07**	**0.13**
		23	0.00				22	0.35	0.14
		24	0.00				23	0.01	0.04
		25	0.00				24	0.01	0.01
							25	0.00	0.00

Clwyd

**Recorded**				**Random**					
AC		SC		AC	MEAN	SD	SC	MEAN	SD
1	0.03	1	0.00	1	0.02	0.00	1	0.00	0.00
2	0.01	4	0.00	2	0.06	0.00	2	0.00	0.00
3	0.02	5	0.00	3	0.02	0.00	4	0.01	0.00
**4**	**0.62**	**9**	**0.00**	**4**	**0.41**	0.01	5	0.00	0.00
**5**	**0.30**	12	0.03	**5**	**0.38**	0.01	**8**	**0.00**	**0.01**
**6**	**0.00**	13	0.01	**6**	**0.06**	0.00	**9**	**0.04**	**0.01**
7	0.02	**14**	**0.65**	7	0.04	0.00	**10**	**0.01**	**0.00**
9	0.00	**15**	**0.03**	8	0.00	0.00	12	0.03	0.01
10	0.00	**18**	**0.10**	9	0.01	0.00	13	0.08	0.07
		**19**	**0.14**	10	0.00	0.00	**14**	**0.42**	**0.07**
		21	0.00				**15**	**0.07**	**0.00**
		22	0.01				**18**	**0.07**	**0.03**
		24	0.02				**19**	**0.22**	**0.04**
		25	0.00				**20**	**0.00**	**0.00**
							21	0.01	0.00
							22	0.01	0.01
							24	0.03	0.01
							25	0.01	0.00

Cumbria

**Recorded**				**Random**					
AC		SC		AC	MEAN	SD	SC	MEAN	SD
1	0.01	1	0.00	1	0.02	0.00	1	0.02	0.00
2	0.01	2	0.00	2	0.03	0.00	2	0.01	0.00
3	0.05	4	0.01	3	0.04	0.00	4	0.04	0.00
**4**	**0.78**	5	0.00	**4**	**0.46**	0.01	5	0.00	0.00
**5**	**0.11**	**8**	**0.00**	**5**	**0.32**	0.00	7	0.00	0.00
**6**	**0.01**	**9**	**0.00**	**6**	**0.05**	0.00	**8**	**0.03**	**0.00**
7	0.02	**10**	**0.00**	7	0.01	0.00	**9**	**0.01**	**0.00**
8	0.00	12	0.01	8	0.01	0.00	**10**	**0.02**	**0.00**
9	0.01	13	0.01	9	0.04	0.00	12	0.02	0.00
10	0.00	**14**	**0.82**	10	0.02	0.00	13	0.03	0.00
		**15**	**0.03**				**14**	**0.49**	**0.00**
		**17**	**0.02**				**15**	**0.10**	**0.00**
		**18**	**0.02**				**17**	**0.06**	**0.00**
		**19**	**0.02**				**18**	**0.03**	**0.00**
		21	0.00				**19**	**0.11**	**0.00**
		22	0.03				21	0.00	0.00
		23	0.00				22	0.02	0.00
		24	0.01				23	0.00	0.00
		25	0.00				24	0.01	0.00
							25	0.00	0.00

Devon

Recorded				Random					
AC		SC		AC	MEAN	SD	SC	MEAN	SD
1	0.03	1	0.00	1	0.04	0.00	1	0.01	0.00
2	0.00	2	0.00	2	0.01	0.00	2	0.00	0.00
3	0.19	4	0.00	3	0.21	0.00	4	0.00	0.00
**4**	**0.73**	5	0.00	**4**	**0.60**	0.00	5	0.00	0.00
**5**	**0.02**	**8**	**0.00**	**5**	**0.07**	0.00	7	0.00	0.00
**6**	**0.00**	**9**	**0.00**	**6**	**0.03**	0.00	**8**	**0.01**	**0.00**
7	0.02	**10**	**0.00**	7	0.03	0.00	**9**	**0.00**	**0.00**
8	0.00	12	0.03	8	0.00	0.00	**10**	**0.02**	**0.01**
9	0.00	13	0.00	9	0.00	0.00	12	0.05	0.01
10	0.00	**14**	**0.80**	10	0.01	0.00	13	0.08	0.20
		**15**	**0.00**				**14**	**0.61**	**0.20**
		**18**	**0.01**				**15**	**0.01**	**0.00**
		**19**	**0.01**				**18**	**0.01**	**0.01**
		21	0.07				**19**	**0.05**	**0.01**
		22	0.03				21	0.08	0.01
		23	0.00				22	0.04	0.01
		24	0.02				23	0.00	0.01
		25	0.00				24	0.02	0.01
							25	0.00	0.00

Aberdeen

LC1	MEAN	SD	LC2	MEAN	SD	LC3	MEAN	SD	LC4	MEAN	SD
4	0.80	0.01	4	0.34	0.01	14	0.89	0.01	9	0.32	0.01
5	0.20	0.01	5	0.08	0.00	15	0.11	0.01	10	0.05	0.00
			6	0.57	0.01				11	0.17	0.01
									14	0.40	0.01
									15	0.05	0.00

Clwyd

LC1	MEAN	SD	LC2	MEAN	SD	LC3	MEAN	SD	LC4	MEAN	SD
4	0.52	0.01	4	0.48	0.01	14	0.87	0.01	9	0.08	0.00
5	0.48	0.01	5	0.45	0.01	15	0.13	0.01	10	0.01	0.00
			6	0.07	0.00				14	0.79	0.01
									15	0.12	0.01

Cumbria

LC1	MEAN	SD	LC2	MEAN	SD	LC3	MEAN	SD	LC4	MEAN	SD
4	0.59	0.01	4	0.55	0.01	14	0.83	0.00	9	0.01	0.00
5	0.41	0.01	5	0.38	0.01	15	0.17	0.00	10	0.03	0.00
			6	0.07	0.00				14	0.80	0.00
									15	0.16	0.00

Devon

LC1	MEAN	SD	LC2	MEAN	SD	LC3	MEAN	SD	LC4	MEAN	SD
4	0.90	0.00	4	0.86	0.00	14	0.99	0.00	9	0.00	0.00
5	0.10	0.00	5	0.10	0.00	15	0.01	0.00	10	0.02	0.00
			6	0.04	0.00				14	0.96	0.00
									15	0.01	0.00
