# Supplementary material for: Disease Prevention versus Data Privacy: Using Landcover Maps to Inform Spatial Epidemic Models
Source: PLoS Comput Biol. 2012 Nov 1;8(11):e1002723. doi: 10.1371/journal.pcbi.1002723 (PMC3486837; doi:10.1371/journal.pcbi.1002723)
Supplement: Table S4 — The mean and 95% confidence intervals of number of IPs, DCs for epidemics seeded in Cumbria with no ring culling, and the optimal ring cull radius RRC (in km) when ring culling is included, for epidemics on the recorded data, the generated data set “Random” and the land cover derived data set LC3. Epidemics are simulated using the UK 2001 dispersal kernel, a kernel with twice the height and half the width of the UK kernel (Kernel 2) and a kernel with half the height and twice the width of the UK kernel (Kernel 3). For the simulated data sets, the range of values of optimal ring cull radii (RRC(min)-RRC(max)) across each group of 100 data sets are shown. (PDF) [file pcbi.1002723.s009.pdf]

		UK 2001 Kernel	Kernel 2	Kernel 3
Recorded Data	IPs	1012 (42-1280)	6 (1-36)	1717 (15-2210)
	DCs	1332 (62-1706)	5 (0-37)	2430 (12-3034)
	R_{RC}	3.6	0.0	6.8
RAN	IPs	90 (6-377)	2 (1-10)	1341 (8-2073)
	DCs	106 (5-446)	1 (0-7)	1759 (10-2658)
	R_{RC} $[R_{RC(min)}\text{-}R_{RC(max)}]$	1.6 (1.0-2.2)	0.0 (0.0-0.0)	5.8 (4.8-6.4)
LC3	IPs	818 (8-1286)	4 (1-24)	1751 (25-2184)
	DCs	1062 (9-1622)	4 (0-23)	2474 (32-3029)
	R_{RC} $[R_{RC(min)}\text{-}R_{RC(max)}]$	3.5 (3.2-3.8)	0.0 (0.0-0.0)	6.6 (6.2-7.0)
