# Supplementary material for: Disease Prevention versus Data Privacy: Using Landcover Maps to Inform Spatial Epidemic Models
Source: PLoS Comput Biol. 2012 Nov 1;8(11):e1002723. doi: 10.1371/journal.pcbi.1002723 (PMC3486837; doi:10.1371/journal.pcbi.1002723)
Supplement: Text S1 — Supplementary information providing details of sensitivity analyses and describing supplementary figures S1 to S3 and supplementary tables S1 to S4. (PDF) [file pcbi.1002723.s010.pdf]

Supplementary Text

In this paper we have shown that the use of highly resolved subclass land use data is able to capture the pattern of spatial clustering of farms in the county of Cumbria. In particular, the absence of farms in the Lake District is clearly visible. Here we examine the ability of the approach to capture the spatial distribution of farms in Devon. From the recorded data (Figure S1a) we see that the highest density of farms is found in the North East and North West of the county, with low densities in the central and South East regions. The center of the county, where no farms are located, is dominated by Dartmoor National Park. If we assume random locations for farms, the resultant density map bears little resemblance to the truth data (Figure S1b). However, when the "Land Cover 3" data set is used, a much better approximation to the truth data is observed, with the absence of farms in the Dartmoor region clearly visible (Figure S1c). For reference, all 10 aggregate classes and 26 subclasses used to categorise Land Cover Map 2000 are listed in table 1 in the main text.

Additionally, to assess the simulated clustering, we measure the average density of farms around each farm at a range of radii, in line with previous work [1]. Significant local clustering is observed in Cumbria (Figure 5 in main text) and is underestimated by both the random data and the LCM 2000 data based upon ACs. SCs perform better, but are still observed to under-represent clustering at small radii. This failure to capture fine scale clustering even when using SCs may provide some explanation for the underestimating of Epidemic Impacts for these data sets. Land Cover Map 2000 captures local clustering much better in Devon, Aberdeenshire and Clwyd where the effect of geographical features upon local clustering is much less marked (Figure S2). However, the results presented above indicate that, for all counties considered in this paper, in the absence of knowledge of farm locations, highly resolved demographic information is vital for making accurate epidemiological and control predictions in the event of an epidemic in livestock.

In order to compare how our land cover type selections and resulting simulated datasets matched both the true farm distribution among land classes, and the distribution of classes within each county, we tabulated these data in Tables S2 and S3. Note that our 'expert opinion' based land class selection worked very well in Cumbria, but becomes less appropriate in Aberdeen, for example, where 60% of farms occurred in the AC land class "Arable and Horticulture" (see Table 1, Table S3). Whether this is due to poor classification in the original land cover data, or our lack of knowledge as to farms being placed in horticultural lands, it is an interesting and influential factor in the data simulation.

Parameter Sensitivity

The model used throughout this paper is an adapted version of the Keeling model that was first used in 2001 to predict the spread of the UK FMD epidemic and to determine policies to control the outbreak [2]. The model has subsequently been used to investigate the impact of vaccination and contiguous culling upon disease control [3,4]. The version used in this paper has also been refitted to the 2001 outbreak (detailed in [5]) with a good agreement found between the model replicates and the 2001 epidemic data at the individual farm scale.

During the 2001 outbreak, the transmission kernel that was used in the Keeling model was estimated from detailed contact tracing performed by local veterinary authorities. However, it is likely that future outbreaks will not spread in the same way. We therefore investigate how significantly our results change when we depart from our original kernel parameters with the introduction of two alternate kernels. We set

$$K_{NEW}=p \times K_{UK}(d_{ij}/q)$$

where K_{UK} is the kernel used during the 2001 outbreak, d_{ij} is the distance between farms i and j and p *and* q are scaling factors that enable us to scale the height and width of the dispersal kernel. We now introduce two new kernels, *Kernel 2* and *Kernel 3*, based upon this formulation. *Kernel 2* is a scaled version of the UK 2001 Kernel with twice the height and half the width ($p=2$, $q=1/2$), simulating a highly transmissible, but very local outbreak). *Kernel 3* is a scaled version of the UK 2001 Kernel with half the height and twice the width ($p=1/2$, $q=2$), simulating a weakly transmitted but highly dispersed outbreak. Our results show that when the majority of disease spread is local (*Kernel 2*), epidemic sizes are very small (fewer than 10 farms). The data sets "Random" underpredict epidemic sizes, whilst epidemic sizes for the data sets LC3 show a good fit to the Recorded data. For all three data sets investigated, the optimal strategy is to employ no ring culling, owing to the small size of the outbreaks (Table S4).

When disease can spread over a large distance (*Kernel 3*), the land cover data sets LC3 provide a good fit to the predicted epidemics on the recorded data – an average of 1717 IPs and 2430 DCs without ring culling and an optimal ring cull radius of 6.8km are predicted when epidemics are simulated using the recorded data compared with an average of 1731 IPs and 2474 DCs without ring culling and a mean optimal ring cull radius of 6.6km when using LC3 data. Significantly, the generated data sets "Random" perform significantly better for *Kernel 3* than for the UK kernel, with 1341 IPs and 1759 DCs on average without ring culling and a mean optimal ring cull radius of 5.8km. This is unsurprising – the local clustering that is not captured by the random location data sets will have a lesser effect when the kernel is flatter and wider (Table S4).

Should a new strain of FMD enter the livestock population the transmissibilities of cattle and sheep may differ significantly from that seen during the 2001 epidemic. We have therefore carried out sensitivity to the transmissibility parameters and the results for T_C (transmissibility of cattle) are shown in Figure S3. Our results for cattle show that the LC3 data sets remain a good fit to the epidemic impact predicted by the recorded data for transmissibilities at least ten times that seen in the 2001 epidemic, whilst the use of random farm locations significantly under predicts epidemic impacts for all values of T_c investigated. Similar results are found when the value of transmissibility of sheep, T_s, is varied. These analyses indicate that the result in the paper, that highly resolved land cover can be used as a proxy for farm locations to predict epidemic sizes and preferred control strategies, is not dependent upon the precise parameters used for the 2001 outbreak and is in fact robust for a range of epidemiological parameter values.

References

1. Tildesley MJ, House TA, Bruhn MC, Curry RJ, O'Neil M, Allpress JL, Smith G, Keeling MJ (2010). Impact of spatial clustering on disease transmission and optimal control. *PNAS* 107: 1041-1046.
2. Keeling MJ, Woolhouse MEJ, Shaw DJ, Matthews L, Chase-Topping ME et al. (2001). Dynamics of the 2001 UK foot and mouth epidemic: stochastic dispersal in a heterogeneous landscape. *Science* **294**: 813-817.
3. Tildesley MJ, Savill NJ, Shaw DJ, Deardon R, Brooks SP et al. (2006). Optimal reactive vaccination strategies for a foot-and-mouth outbreak in Great Britain. *Nature* **440**: 83-86.
4. Tildesley MJ, Bessell PR, Keeling MJ, Woolhouse MEJ (2009). The role of pre-emptive culling in the control of Foot-and-Mouth Disease. *Proc. Roy. Soc. B* **276**: 3239-3248.
5. Tildesley MJ, Deardon R, Savill NJ, Bessell PR, Brooks SP et al. (2008) Accuracy of models for the 2001 foot-and-mouth Epidemic. *Proc. Roy. Soc. B* **275**: 1459-1468.
